# Avian Influenza Virus Glycoproteins Restrict Virus Replication and Spread through Human Airway Epithelium at Temperatures of the Proximal Airways

**DOI:** 10.1371/journal.ppat.1000424

**Published:** 2009-05-15

**Authors:** Margaret A. Scull, Laura Gillim-Ross, Celia Santos, Kim L. Roberts, Elena Bordonali, Kanta Subbarao, Wendy S. Barclay, Raymond J. Pickles

**Affiliations:** 1 Cystic Fibrosis/Pulmonary Research and Treatment Center, University of North Carolina at Chapel Hill, Chapel Hill, North Carolina, United States of America; 2 Department of Microbiology and Immunology, University of North Carolina at Chapel Hill, Chapel Hill, North Carolina, United States of America; 3 Laboratory of Infectious Diseases, Respiratory Viruses Section, National Institute of Allergy and Infectious Diseases, National Institutes of Health, Department of Health and Human Services, Bethesda, Maryland, United States of America; 4 Department of Virology, Division of Investigative Science, Faculty of Medicine, Imperial College London, St. Mary's Campus, London, United Kingdom; 5 Department of Biostatistics, University of North Carolina at Chapel Hill, Chapel Hill, North Carolina, United States of America; Erasmus Medical Center, The Netherlands

## Abstract

Transmission of avian influenza viruses from bird to human is a rare event even though avian influenza viruses infect the ciliated epithelium of human airways *in vitro* and *ex vivo*. Using an *in vitro* model of human ciliated airway epithelium (HAE), we demonstrate that while human and avian influenza viruses efficiently infect at temperatures of the human distal airways (37°C), avian, but not human, influenza viruses are restricted for infection at the cooler temperatures of the human proximal airways (32°C). These data support the hypothesis that avian influenza viruses, ordinarily adapted to the temperature of the avian enteric tract (40°C), rarely infect humans, in part due to differences in host airway regional temperatures. Previously, a critical residue at position 627 in the avian influenza virus polymerase subunit, PB2, was identified as conferring temperature-dependency in mammalian cells. Here, we use reverse genetics to show that avianization of residue 627 attenuates a human virus, but does not account for the different infection between 32°C and 37°C. To determine the mechanism of temperature restriction of avian influenza viruses in HAE at 32°C, we generated recombinant human influenza viruses in either the A/Victoria/3/75 (H3N2) or A/PR/8/34 (H1N1) genetic background that contained avian or avian-like glycoproteins. Two of these viruses, A/Victoria/3/75 with L226Q and S228G mutations in hemagglutinin (HA) and neuraminidase (NA) from A/Chick/Italy/1347/99 and A/PR/8/34 containing the H7 and N1 from A/Chick/Italy/1347/99, exhibited temperature restriction approaching that of wholly avian influenza viruses. These data suggest that influenza viruses bearing avian or avian-like surface glycoproteins have a reduced capacity to establish productive infection at the temperature of the human proximal airways. This temperature restriction may limit zoonotic transmission of avian influenza viruses and suggests that adaptation of avian influenza viruses to efficient infection at 32°C may represent a critical evolutionary step enabling human-to-human transmission.

## Author Summary

Influenza type A viruses are endemic in aquatic birds but can cross the species barrier to infect the human respiratory tract. While transmission from birds to humans is rare, the introduction of novel avian influenza viruses into immunologically naïve human populations has significant pandemic potential. Avian influenza viruses are adapted for growth at 40°C, the temperature of the avian enteric tract. However, the human proximal airways, the likely site of initial inoculation by influenza viruses, are maintained at a cooler temperature (32°C), suggesting that zoonotic transmission may be limited by temperature differences between the two hosts. Using an *in vitro* model of human ciliated airway epithelium, we show that avian influenza viruses grow well at 37°C, a temperature reflective of distal airways, but are restricted for infection at 32°C. A panel of genetically manipulated human influenza viruses possessing avian or avian-like surface glycoproteins were also restricted at 32°C, but not 37°C, suggesting that avian virus glycoproteins are not adapted for efficient infection at the temperature of the proximal airways. Thus, avian influenza virus infection is restricted in the human proximal airways due to the cooler temperature of this region, thus limiting the likelihood of zoonotic and subsequent human-to-human transmission of these viruses.

## Introduction

Influenza viruses circulating in the human population are predominately type A and B, with type A being more common [1]. All influenza type A viruses originate from aquatic birds and successful introduction of these avian viruses into the human population, by either direct adaptation or reassortment with already circulating human viruses, has led to influenza pandemics of historical significance (reviewed in [2]–[4],[5]). Still, documented evidence of transmission of avian influenza viruses directly from birds to humans is rare, partly because species barriers restrict avian influenza virus infection of the epithelial cells of the human respiratory tract, the primary site of influenza virus infection and spread.

Influenza A viruses possess a hemagglutinin (HA) attachment protein that binds sialic acid residues to facilitate infection of target epithelial cells. The HA of human influenza viruses preferentially binds to terminal sialic acid (SA) residues with α2,6 linkages, whereas avian influenza viruses preferentially bind to SA with α2,3 linkages [6]–[9]. The prevalence of α2,6 SA but paucity of α2,3 SA in the human respiratory tract has been considered to restrict infection by avian influenza viruses [10]. Recent reports, however, have detected significant levels of α2,3 SA on human airway epithelium both *in vitro* and *ex vivo*, including in nasopharyngeal and tracheobronchial tissue [11]–[14]. This SA distribution also correlated with avian influenza virus infection *in vitro* and *ex vivo* and raised the possibility that avian viruses could infect the upper airways *in vivo*. Therefore, although it is universally accepted that human-to-human transmission of avian influenza viruses requires adaptation of HA to switch from α2,3 to α2,6 SA usage, the cumulative data published to date indicate that SA linkages and their respective distribution in the human airways are not the sole barrier to avian influenza virus infection [15]–[17]. Other host factors and viral genes are likely also important determinants of infectivity.

One such host factor that may limit zoonotic transmission is the difference in host temperatures between avian and human tissues that are susceptible to influenza virus infection. Avian influenza viruses are adapted for replication in the avian enteric tract at 40–41°C. While the surface temperatures of the human respiratory tract are variable, a temperature gradient clearly exists in which the surface temperature of the proximal large airways (i.e., nasal and tracheal) average 32+/−0.05°C while temperatures of the smaller, distal airways (i.e., bronchioles) are closer to that of the core body temperature, 37°C [18],[19]. While multiple transmission routes have been described for influenza viruses, the proximal airways likely represent a predominant site for human influenza virus inoculation as they provide a large exposed surface area of virus-susceptible epithelial cells [20]. These cells are directly accessible by large droplet aerosols and by way of digital inoculation of the nasopharynx and conjunctival mucosa [12],[21]. Inefficient infection by avian influenza viruses, even in the presence of α2,3-linked SA, may be due to the cooler temperature of the proximal airways compared to that of the distal airways/lung regions where H5N1 avian influenza viruses appear to replicate efficiently [22].

Avian influenza viruses are attenuated at temperatures below 37°C and cold sensitivity of avian viral RNA replication in cell lines was linked to the presence of a glutamic acid at amino acid 627 in the avian virus polymerase subunit, PB2, instead of a lysine in the human virus PB2 [23]. Lysine substitution at residue 627 of H5N1 viruses improved virus replication in mice [24]. In addition to PB2, work utilizing human-avian reassortant viruses in MDCK cells provided initial evidence that avian glycoproteins, HA and neuraminidase (NA), may mediate temperature-dependent effects on viral growth [25]. To our knowledge, other viral genes have not been well characterized, nor the HA and NA further evaluated, in their contribution to temperature sensitivity of avian influenza viruses.

To characterize the temperature dependency of avian vs. human influenza viruses in a relevant model of the target cell types of the human airways, we utilized an *in vitro* model of human ciliated airway epithelium (HAE). This model closely mimics the morphological and physiological features of the human airway epithelium *in vivo* and has been previously used to investigate infection by diverse respiratory viruses [26]–[30]. In humans, ciliated airway epithelium is present throughout the airways, extending from the nasal cavity and large proximal airways into the distal bronchiolar airway regions. Previously, we have shown that both human and avian influenza viruses replicate well in HAE and that human and avian influenza virus cell tropism correlates with the respective distribution of the specific sialic acid linkages [13]. However, these previous studies were conducted at 37°C, reflecting conditions encountered in the distal airways [13]. Others have also utilized these airway cell systems to characterize influenza virus replication of wild-type and recombinant viruses at 35°C [14],[31],[32]. In the present study, we utilize the HAE model, in combination with influenza virus reverse genetics, to investigate the influence of temperature on human and avian influenza virus infection, replication and spread. We demonstrate that, compared to human influenza viruses, avian influenza viruses are severely restricted for infection of human airway epithelium at the temperature of the human proximal airways. Then, using different strategies to ‘avianize’ human influenza viruses, we show that the temperature restriction of avian viruses is closely associated with the avian HA and NA glycoproteins.

## Results

### Human and avian influenza virus infection of human ciliated airway epithelium at 32°C and 37°C

We and others have previously shown that human and avian influenza viruses infect and replicate in HAE [13],[14],[31]. Since our previous experiments were performed at 37°C, a temperature reflective of human distal airways, we have now compared human and avian influenza virus infection and growth in HAE at temperatures reflective of the proximal airways (32–33°C) and distal airways (37°C). HAE were inoculated at either 32°C or 37°C with a low multiplicity of infection (MOI; 0.01) of a representative human virus, A/Victoria/3/75 (H3N2), or an avian influenza isolate, A/Dk/Eng/62 (H4N6). Virus growth and spread throughout the epithelium at the two temperatures was measured and compared over time and infection further characterized with respect to virus-induced cytopathic effects (CPE).

**Figure 1:**
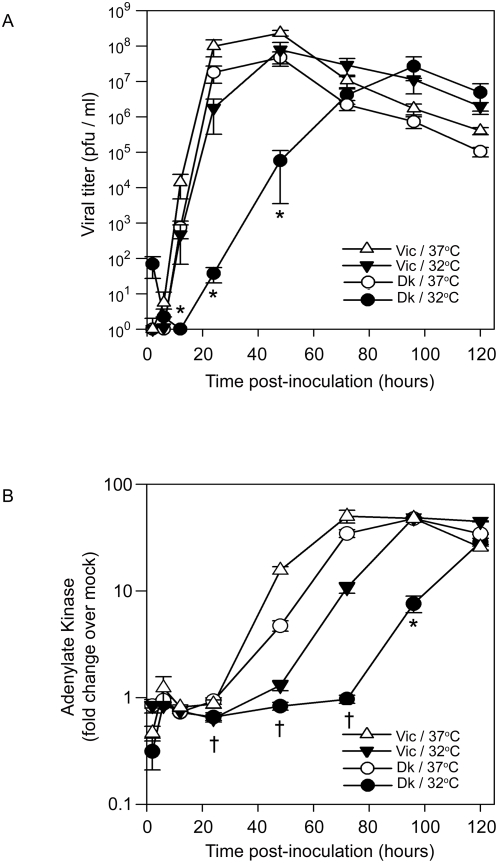
Infection of HAE by avian, but not human, influenza viruses is restricted at temperatures of the proximal airways. (A) Comparison of multi-cycle virus growth in HAE inoculated with either A/Victoria/3/75 at 32°C (*closed triangles*) or 37°C (*open triangles*) and A/Dk/Eng/62 at 32°C (*closed circles*) or 37°C (*open circles*) both at MOI∼0.01. Apical viral titers at times shown were determined by standard plaque assay on MDCK cells. Data shown represents the mean titer +/−standard error (SE; n = 3–10 cultures). (B) Adenylate kinase activity released into the apical compartment of HAE over time after inoculation with A/Victoria/3/75 or A/Dk/Eng/62 at 32°C and 37°C as a measure of viral-induced CPE. Data shown represents the mean fold change over adenylate kinase activity derived from mock-inoculated HAE +/−SE (n = 3–8). Significance is noted (*p<0.05) where viral titers or AK levels obtained for A/Dk/Eng/62 at 32°C were statistically different from all other titers/AK measurements (Dk/37°C, Vic/32°C and Vic/37°C) at that particular time point. Significance is noted (^†^p<0.05) where AK levels obtained for A/Dk/Eng/62 at 32°C and 37°C were statistically different.

At the temperature of the distal airways (37°C), the growth kinetics and mean peak titers of A/Victoria/3/75 and A/Dk/Eng/62 reached 2.3×10^8^ pfu/ml and 4.7×10^7^ pfu/ml, respectively, by 48 hours post-inoculation (hrs pi) (Figure 1A). At the temperature of the proximal airways (32°C), A/Victoria/3/75 showed a modest delay in replication but still reached maximal titer of 7.8×10^7^ pfu/ml by 48 hrs pi. In contrast, A/Dk/Eng/62 grew very slowly, with yields at time points up to 48 hrs pi reduced by 3 to 5 logs compared to growth for this virus at 37°C or A/Victoria/3/75 at either temperature.

In comparison to 48 hr titers, A/Victoria/3/75 titers at both temperatures and A/Dk/Eng/62 titers at 37°C were reduced at 72 hr pi and every time point thereafter, indicating reduced progeny virus production. A loss of titer was also observed for A/Dk/Eng/62 at 32°C, but not before 120 hrs pi. To determine if loss of titer after reaching maximum levels correlated with increased CPE, we quantified adenylate kinase (AK) release by dead/dying cells into the apical compartment as a sensitive and global measure of cytotoxicity across the entire epithelial cell culture surface. Figure 1B indicates that substantial increases in AK levels, indicative of the onset of CPE, are first detected at 48 hrs pi for A/Victoria/3/75 at 32°C and 37°C and A/Dk/Eng/62 at 37°C. This induction of AK coincided with peak viral titer for these viruses under these conditions (compare Figure 1A and 1B) and suggested that the loss of titer correlated with the onset of CPE. Increasing levels of AK between 48 and 96 hrs pi were directly associated with continually decreasing viral titers, further supporting this claim.

**Figure 2:**
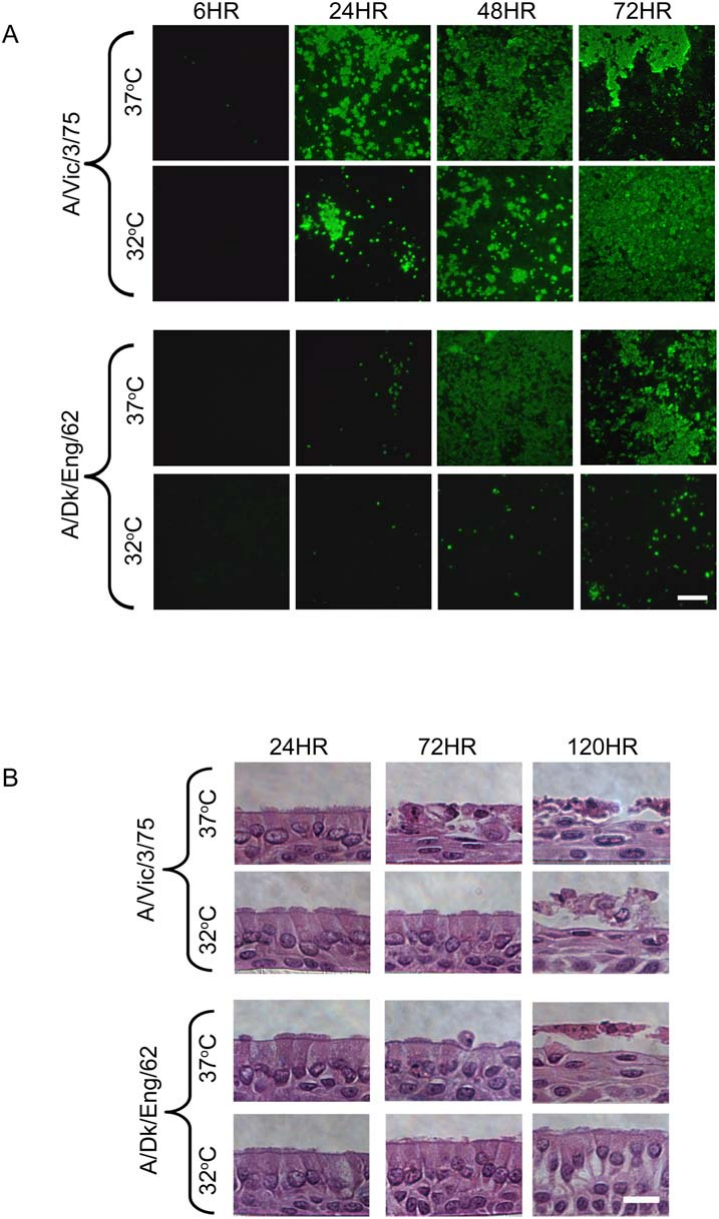
Spread and histopathology of avian and human influenza viruses in HAE at temperatures of the proximal and distal airway. (A) Representative *en face* photomicrographs of HAE inoculated with either A/Victoria/3/75 or A/Dk/Eng/62 at 32°C or 37°C, fixed at 6, 24, 48 and 72 hrs pi and stained for viral nucleoprotein (*green*) to determine numbers of cells infected. Scale bar equals 100 µm. (B) Representative histological cross-sections of HAE at 24, 72 and 120 hrs after inoculation with A/Victoria/3/75 or A/Dk/Eng/62 at 32°C or 37°C. H&E counterstain. Scale bar equals 20 µm.

A relationship between the kinetics of virus growth in HAE and the level of CPE also suggested that CPE was a consequence of viral replication. This assertion is supported by the fact that trends in viral titers at a given time point are mirrored in AK levels detected 48 hrs later (e.g., compare viral titers at 48 hr pi (Figure 1A) to AK measurements taken at 96 hr pi (Figure 1B)). Since viral titer and AK levels could be related to the numbers of cells infected and/or the degree of virus replication within individual cells we compared titers of human and avian influenza viruses (Figure 1A) to the numbers of cells infected by each virus at the two temperatures over time. Immunodetection of viral antigen in inoculated HAE showed that human and avian influenza virus antigen was not detected 3 hrs pi, indicating that levels of antigen in residual viral inocula were below the limit of antibody detection (data not shown). For A/Victoria/3/75, a few isolated cells were positive for viral antigen by 6 hrs pi at 37°C, but by 24 hrs pi considerable numbers of antigen-positive cells were detected (Figure 2A). In agreement with our growth curves in Figure 1A, A/Victoria/3/75 infected slightly fewer cells at 32°C compared to 37°C at 24 hrs pi, but importantly, A/Victoria/3/75 spread efficiently within the epithelium at both temperatures and differences in infection at early time points became less significant over time (Figure 2A).

In contrast to A/Victoria/3/75, A/Dk/Eng/62 antigen was detected in only a few cells 24 hrs pi at either temperature. However, it should be noted that antigen-positive cells in *en face* images are viewed linearly (Figure 2A) whereas viral titers are shown on a logarithmic scale (Figure 1A). Thus, an apparently small difference in titer as is seen at 24 hrs pi between A/Victoria/3/75 and A/Dk/Eng/62 at 37°C may correspond to a larger difference in the number of cells positive for viral antigen. While our staining also confirmed previous data that avian influenza viruses infect fewer human airway epithelial cells in comparison to human influenza virus at 37°C (Figure 2A; [13]), the limited extent of A/Dk/Eng/62 antigen positive cells at 37°C by 24 hr pi was still unexpected given that titers at this time were slightly greater than those for A/Victoria/3/75 at 32°C. Whether this represents a difference in yield of infectious virus per infected cell between human and avian viruses is presently not clear. Overall, A/Dk/Eng/62 grew and spread well at 37°C, but was severely restricted for growth at 32°C and antigen positive cells were barely detectable before 48 hr pi for this virus at lower temperature.

HAE cultures infected with A/Victoria/3/75 at either 32°C or 37°C and A/Dk/Eng/62 at 37°C viewed *en face* exhibited loss of integrity of the epithelium although the extent of injury and time of onset varied (Figure 2A). Further evaluation of histological cross-sections indicated that A/Victoria/3/75 infection at 37°C, which had the highest and earliest induction of AK, resulted in the earliest evidence of morphological injury at 72 hrs pi. HAE infected with A/Victoria/3/75 at 32°C or 37°C or A/Dk/Eng/62 at 37°C all showed desquamation of the superficial layer of columnar epithelial cells with basal epithelial cells remaining attached to the matrix support by 120 hrs pi (Figure 2B). Similar cytopathology has been reported for A/Udorn/307/72 influenza virus infection of HAE *in vitro* and for clinical human influenza virus infection *in vivo* [29],[33]. The detection of AK in apical washes of A/Dk/Eng/62-infected HAE at 32°C suggested that this virus did eventually compromise cellular integrity at the lower temperature, but dramatic morphological effects were not seen at least for up to 120 hrs (Figure 1B and 2B). It should be noted, however, that at 120 hrs pi, A/Dk/Eng/62-infected HAE at 32°C did display some morphological characteristics different from uninfected and infected HAE at earlier time-points. Preliminary assessment indicates that expansion of lateral spaces between the columnar epithelial cells had occurred. Although we do not know the significance of these morphological changes, we speculate these observations are the initiation of CPE that will ultimately result in similar cellular injury as seen for this virus at 37°C and human viruses at both temperatures.

In sum, for both viruses at both temperatures, detection of maximal numbers of antigen-positive cells correlated with high titers (compare Figure 1A and 2A) and increasing CPE (Figure 1B). By 72 and 120 hrs pi considerable loss of cells from the culture was evident and this correlated with the drop off in viral titers at these time points (Figure 1A). Thus, we conclude that in the context of maximal infection in which there were no additional target cells available for infection within the finite surface area of the HAE culture, ongoing replication in antigen-positive cells shown at 48 and 72 hrs pi resulted in increased cell death. This CPE led to a reduction in the number of viable, virus-producing cells and in turn, to a reduction in progeny virus. Although A/Dk/Eng/62 induced CPE when sufficient titers were generated at 37°C, one consequence of restricted replication of this avian influenza virus at 32°C was a reduction in overt CPE in HAE, even at later time points associated with considerable viral titers.

### Avian influenza virus restriction at 32°C is independent of avian virus strain

**Figure 3:**
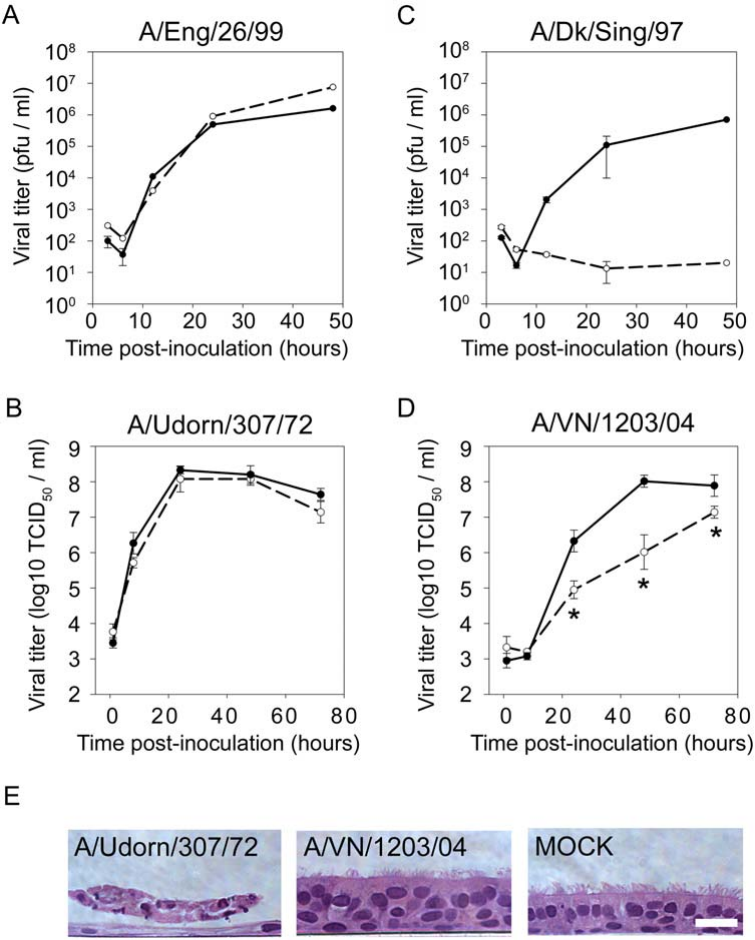
Temperature-dependent growth of different serotypes of influenza viruses in HAE. Multi-step growth kinetics of (A) human influenza virus A/Eng/26/99 or (C) avian influenza virus A/Dk/Sing/97 (MOI∼0.1) at 32°C (*open circles*, *dashed line*) or 37°C (*closed circles*, *solid line*) in HAE +/−SE (n = 3 cultures). Multi-step growth kinetics in HAE inoculated with an MOI∼0.03 of (B) A/Udorn/307/72 (H3N2) or (D) A/VN/1203/04 (H5N1) at 33°C (*open circles*, *dashed line*) or 37°C (*closed circles*, *solid line*). Data represents mean titer across two different donors, each performed in duplicate +/−SE. Viral titers were determined by plaque assay in (A) and (B) and by TCID_50_ assay for (C) and (D). No significant differences in growth between temperatures were found for either A/Eng/26/99 or A/Udorn/307/72. A/VN/1203/04 was significantly restricted for growth at 24, 48 and 72 hrs pi (*p<0.05). (E) Representative histological cross-sections of HAE infected for 72 hrs at 37°C with A/Udorn/307/72 or A/VN/1203/04 and compared to mock-inoculated HAE. H&E counterstain. Scale bar equals 20 µm.

To determine whether other avian, but not human, influenza viruses display temperature dependent phenotypes, we performed multi-step growth curves with more human H3N2 isolates (A/Eng/26/99 and A/Udorn/307/72) and A/Dk/Sing/97, an avian isolate of different subtype (H5N3). Growth of both human-derived influenza viruses tested, A/Eng/26/99 (H3N2) and A/Udorn/307/72 (H3N2), was not significantly different between 32/33°C and 37°C (Figure 3A and 3B). Indeed, these two additional human influenza virus strains showed even less difference in titer between temperatures than was determined for A/Victoria/3/75.

Assessment of growth of avian influenza virus, A/Dk/Sing/97 (H5N3), over a 48 hr time course at 37°C showed similar growth kinetics to that of A/Eng/26/99 (H3N2), reaching titers of 7×10^5^ pfu/ml and 1.6×10^6^ pfu/ml, respectively (Figure 3A and 3C). In contrast, at 32°C, A/Dk/Sing/97 (H5N3) failed to grow at all (Figure 3C). Clearly, the restriction of A/Dk/Sing/97 at 32°C compared to 37°C was an even more striking phenotype than A/Duck/Eng/62. As the avian influenza virus strains used in this study were selected at random, with no selection for a temperature-dependent phenotype, we propose that low temperature restriction of avian influenza viruses, but not human influenza viruses, may be broadly characteristic of avian influenza viruses. The extent of restriction, however, may be variable between different virus strains.

Since the avian virus isolates used in these experiments are neither derived from samples obtained from humans nor passaged in human cells *in vitro*, we next investigated whether growth attenuation at low temperatures would be retained in a highly pathogenic H5N1 (A/VN/1203/04) influenza virus isolated from a fatal human case [34]. We compared infection kinetics of H5N1 (A/VN/1203/04) at 33°C and 37°C on HAE using A/Udorn/307/72 in parallel cultures as a human influenza virus control. As described above, A/Udorn/307/72 grew with similar kinetics at 33°C and 37°C (Figure 3B). A/VN/1203/04, however, exhibited slower replication kinetics at 33°C when compared to that for 37°C (Figure 3D). Indeed, titers were significantly decreased at 33°C vs. 37°C at 24, 48 and 72 hrs pi. In addition, only at 37°C did A/VN/1203/04 approach similar peak titers as the human A/Udorn/307/72 virus by the end of the 72 hr time course (Figure 3D). Histological analyses of A/VN/1203/04-infected HAE at either temperature showed absence of obvious CPE in sharp contrast to A/Udorn/307/72 that obliterated the epithelium by 72 hrs pi (Figure 3E). The lack of obvious CPE after H5N1 infection contrasts reports that H5N1 induced extensive apoptosis in mammalian airway cells [35],[36]. The fact that we did not observe obvious CPE with this highly pathogenic virus warrants further investigation but is in line with the limited cell damage shown following infection with A/Dk/Eng/62 for 72 hrs (Figure 2B). In sum, using diverse examples of human and avian influenza viruses we have shown that avian influenza viruses, but not human influenza viruses, are restricted for infection and growth in HAE at the lower temperature of 32°C.

### ‘Avianization’ of human virus polymerase restricts growth in HAE at both 32°C and 37°C

**Figure 4:**
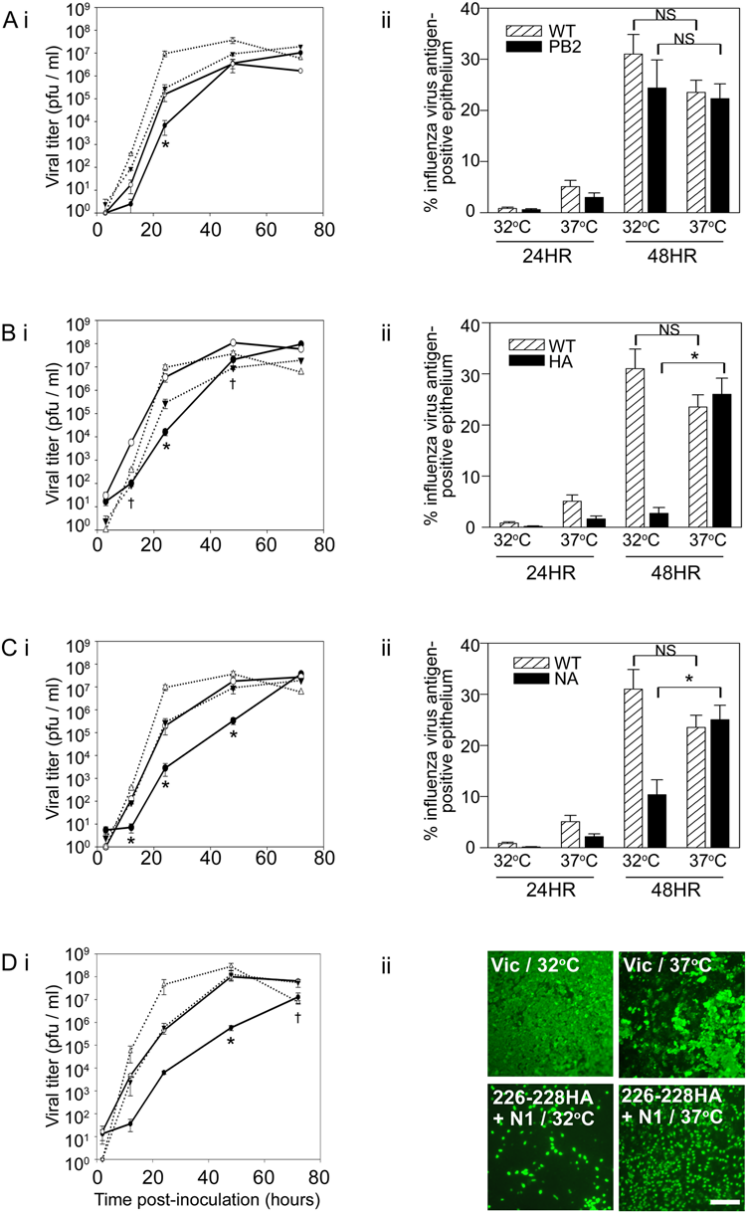
Comparison of replication kinetics and spread of A/Victoria/3/75 with an avianized PB2, HA and/or NA to wild-type virus. Multi-step growth kinetics in HAE inoculated with (Ai) PB2 polymerase mutant (K627E), (Bi) HA (L226Q, S228G) mutant, (Ci) Vic+Chick N1 reassortant virus, (Di) Vic (226–228)HA+Chick N1 at 32°C (*solid line*, *closed circles*) or 37°C (*solid line*, *open circles*). Wild-type A/Victoria/3/75 growth curves at 32°C (*closed triangles*) and 37°C (*open triangles*) are repeated in each panel and shown as dotted lines in (Ai), (Bi), (Ci) and (Di) for comparison. Data represent mean titer across 3–8 cultures +/−SE. Significance is noted (*p<0.05) where viral titer obtained for the mutant or reassortant virus at 32°C was statistically different from all other viral titers (mutant/37°C, wild-type/32°C and wild-type/37°C) at that time point. Significance is noted (^†^p<0.05) where viral titers obtained for the mutant / reassortant virus at 32°C and 37°C were statistically different. Quantification of numbers of cells infected (determined by *en face* staining for viral nucleoprotein) in HAE at 24 and 48 hrs pi at 32°C and 37°C for (Aii) PB mutant virus, (Bii) HA mutant virus and (Cii) N1 reassortant virus. Data obtained in parallel for wild-type A/Victoria/3/75 is repeated in each graph (*striped bars*) for comparison to the mutant (*solid bars*). Data shown represents the mean of the percentage of influenza virus antigen-positive epithelium across 10 different fields +/−SE. Differences in viral antigen positive epithelium between temperatures for each virus at 48 hrs pi is noted as significant (*p<0.05) or insignificant (NS). A one-way ANOVA model showed no significant differences between the wild-type virus and PB2 mutant at 32°C and 37°C at 48 hrs pi. (Dii) Representative *en face* photomicrographs of HAE inoculated with A/Victoria/3/75 or Vic (226–228)HA+Chick N1 at either 32°C or 37°C and stained for viral nucleoprotein (*green*) to determine numbers of cells infected 72 hrs pi. Scale bar represents 100 µm.

Previously, the polymerase subunit PB2 has been shown to play an important role in host range restriction of avian influenza viruses in mammalian cells [37]–[39]. In influenza virus strains that circulate in humans, amino acid residue 627 in PB2 is a lysine, whereas in the majority of avian strains it is a conserved glutamic acid residue. The presence of glutamic acid at PB2 627 (avian-like) has been reported to account for the lower replication of avian influenza strains in mammalian cells and has been linked with reduced polymerase activity at lower temperature (33°C) in some cell systems [23],[24]. To assess the potential impact of this PB2 amino acid residue in restriction of avian influenza viruses at 32°C, we generated a recombinant A/Victoria/3/75 virus containing the PB2 K627E mutation and compared its growth with that of the isogenic wild-type virus in HAE at 32°C and 37°C. The K627E mutation resulted in restriction of the virus at both temperatures (Figure 4Ai), and although titer at 32°C was 1.3 logs lower than at 37°C at 24 hrs pi, this difference was no greater than the differences in growth for wild-type virus at these temperatures (1.5 logs; Figure 4Ai). Moreover, at the later time points analyzed, 48 and 72 hrs pi, the PB2 mutant did not show a significant difference in titer between the two temperatures. These data indicate that the K627E mutant virus was restricted for growth in HAE but that restriction was not temperature-dependent. Indeed, quantification of the numbers of infected cells identified by *en face* staining revealed that the K627E mutant virus infected a similar percentage of cells compared to wild-type virus at 24 hrs pi (Figure 4Aii) and that the mutant was capable of spread since new cells were infected by 48 hrs with similar kinetics to that of wild-type A/Victoria/3/75 at both 32°C and 37°C (Figure 4Aii). Statistically, there was no difference between the wild-type and PB2 mutant viruses at either 32°C or 37°C at 48 hrs pi with respect to percent influenza virus-antigen positive epithelium. Together, these data suggest that the amino acid residue at PB2 627 influences viral fitness in HAE, but does not confer to a human virus the temperature-dependent phenotype of avian influenza virus infection in human ciliated airway epithelium.

### Human influenza viruses with avian-like glycoproteins display restricted replication and spread at 32°C in HAE

Our initial phenotype indicated that A/Dk/Eng/62 was restricted in its ability to spread from cell to cell within the epithelium at 32°C (Figure 2A). Several events in the viral life cycle that are critical for spread, including release of progeny virions from previously infected cells and attachment and entry into new target cells, are mediated by influenza virus glycoproteins. Thus, we hypothesized that glycoprotein function could be responsible for the restricted infection of HAE by avian influenza viruses at the lower temperature of 32°C. To test whether HA and/or NA contributed to the restricted phenotype of avian influenza viruses at 32°C, we used reverse genetics to generate mutant viruses genetically altered to confer avian virus-like glycoprotein specificities on the A/Victoria/3/75 background. First, mutations in HA previously shown to switch sialic acid usage from α2,6 to α2,3 linkages (L226Q, S228G) [40] were introduced to generate the Vic-226-228HA virus. Second, we generated a reassortant virus in which the Victoria NA was replaced by that of the avian virus A/Chick/Italy/1347/99 to generate Vic+Chick N1.

We again compared virus replication and spread of the recombinant viruses to that of wild-type A/Victoria/3/75 at the two temperatures. As stated above, replication measured for the wild-type virus was slightly compromised at lower temperature, noticeable at 24 hrs pi. Restriction at this time point was also observed during infection of HAE with Vic-226-228HA, as it had been for the PB2 mutant virus. Specifically, a 2.5 log decrease in virus growth was determined for Vic-226-228HA at 32°C compared to 37°C at the 24 hr time point (Figure 4Bi). However, unlike the PB2 mutant virus, the difference between replication at 32°C and 37°C for Vic 226-228HA was also significant at the 48 hour time point. Moreover, this mutant virus with avian virus-like sialic acid usage spread less efficiently than wild-type at 32°C so that by 48 hrs pi the number of virus antigen-positive cells was significantly different (Figure 4Bii). In contrast, at 37°C, Vic-226-228HA infected similar numbers of cells as the wild-type virus by 48 hrs; indeed, the mutant virus was able to spread significantly more efficiently at the higher temperature (Figure 4Bii).

Similarly, the reassorted virus Vic+Chick N1 displayed a 2 log decrease in viral titer in HAE at 32°C compared to 37°C at 24 hrs pi. Although this difference was not appreciably greater than the difference in titer between temperatures for either wild-type virus or the PB2 mutant, Vic+Chick N1, unlike wild-type A/Victoria/3/75 and Vic 627PB2, maintained the ∼2-log difference in growth at 48 hrs pi (Figure 4Ci), suggesting this virus was more restricted at the cooler temperature. Quantification of numbers of infected cells illustrated that, like Vic-226-228HA, Vic+Chick N1 was restricted for spread at 32°C which was significant at 48 hrs, but was capable of spread similar to wild-type A/Victoria/3/75 at 37°C (Figure 4Cii). Together these data suggest that avianizing either the HA or NA glycoprotein of an otherwise human influenza virus limits spread and subsequent infection at 32°C compared to 37°C.

We next generated a recombinant influenza virus containing both the 226-228HA and Chick N1 and tested infection and growth in HAE at 32°C and 37°C in comparison to wild-type A/Victoria/3/75. At 24 hrs pi, the double glycoprotein-altered virus exhibited similar restriction as observed for the other viruses. Nonetheless, an overall evaluation of the double glycoprotein-altered virus suggested that as infection proceeded, this virus was profoundly restricted at 32°C compared to 37°C (Figure 4Di), exhibiting >2 log reduction in titer at 48 hrs. Notably, titers for the wild-type virus differed by less than 0.5 logs between temperatures at this time point. Furthermore, the double glycoprotein-altered virus was still significantly restricted at 72 hrs pi when titers at 32°C were compared to those at 37°C. The level of restriction observed for the double mutant was greater than that observed for either virus containing each of these mutations/substitutions individually. Moreover, analysis of viral antigen positive cells at 72 hrs by *en face* staining of infected HAE indicated compromised spread of Victoria (226-228HA)+Chick N1 which was more severe at 32°C than 37°C (Figure 4Dii).

Determination of CPE during these experiments revealed that the double glycoprotein-avianized virus only produced CPE at 72 hrs pi when experiments were performed at 37°C, whereas wild-type human virus produced CPE earlier and at both temperatures (data not shown). These data are consistent with the levels of CPE observed for A/Dk/Eng/62 (H4N6) and A/Victoria/3/75 (H3N2) in our initial studies (Figure 1B) and suggest that altering the human virus glycoproteins to avian virus-like characteristics has profound effects on infection, spread and CPE in the environment of the human ciliated airway epithelium.

### Avian influenza virus glycoproteins dictate cell tropism and restrict growth of virus in HAE at 32°C

**Figure 5:**
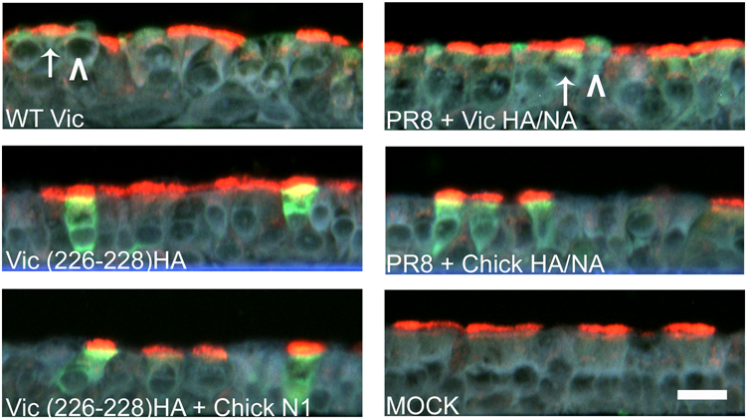
Cell tropism of human, avian and avianized viruses in HAE. Representative cross-sections of inoculated HAE, fixed 24 hrs pi, were probed for viral antigen (NP; green) and α−acetylated tubulin, a marker for ciliated cells (red). Notably, the staining pattern for wild-type A/Victoria/3/75 was identical to that of PR8+Vic HA/NA. Arrows mark ciliated cells infected with either wild-type A/Victoria/3/75 or PR8+Vic HA/NA; arrow-head denotes non-ciliated cells infected by these viruses. These data indicate that viruses with Victoria glycoproteins were able to infect both cell types previously shown to express α2,6 SA [13]. Viral antigen was detected only in ciliated cells in cultures inoculated with Vic-226-228HA (in the Victoria background with either endogenous N2 or avian N1 or PR8+Chick HA/NA). Scale bar equals 20 µm.

One potential caveat of the recombinant viruses with avianized HA and/or NA utilized in our previous analysis was that they contained HA and NA pairs that had not co-evolved. To eliminate the possibility that the restriction we observed with these recombinant viruses was due to an imbalance between the activities of the surface glycoproteins that were not evolutionarily optimized, we next generated reassorted influenza viruses on a common genetic background, possessing human or avian glycoproteins with co-evolved pairings. This was achieved using human recombinant A/PR/8/34 (H1N1) in which the wild-type H1 and N1 glycoproteins were replaced by the H3 and N2 glycoprotein pair from A/Victoria/3/75 (generating PR8+Vic HA/NA) or the H7 and N1 glycoprotein pair from A/Chick/Italy/1347/99 (generating PR8+Chick HA/NA, previously termed RD3) [41]. Since we and others have shown differential cell-type tropism between human and avian influenza virus in HAE [13],[14], we next determined if avianizing the human virus HA by mutation or substitution (in the presence or absence of an avian NA) recapitulated the cell-type tropism exhibited by wholly avian influenza viruses in HAE. As shown by immunofluorescent detection in histological sections of infected HAE, PR8 containing A/Victoria/3/75 glycoproteins infected both ciliated and non-ciliated cells in HAE with a tropism similar to wild-type A/Victoria/3/75 (Figure 5). In contrast, A/Victoria/3/75 with two avian-like amino acid substitutions in HA and PR8+Chick HA/NA only infected ciliated cells, a tropism that was mirrored by wholly avian virus [13],[14]. These data clearly show that the ciliated cell tropism of avian influenza viruses is dictated by properties of the viral glycoproteins. These results correlate with the known increased sialic acid binding preference of avian HA for α2,3-linked SA, and to the presence of α2,3-linked SA on ciliated cells in HAE [8],[13],[14].

**Figure 6:**
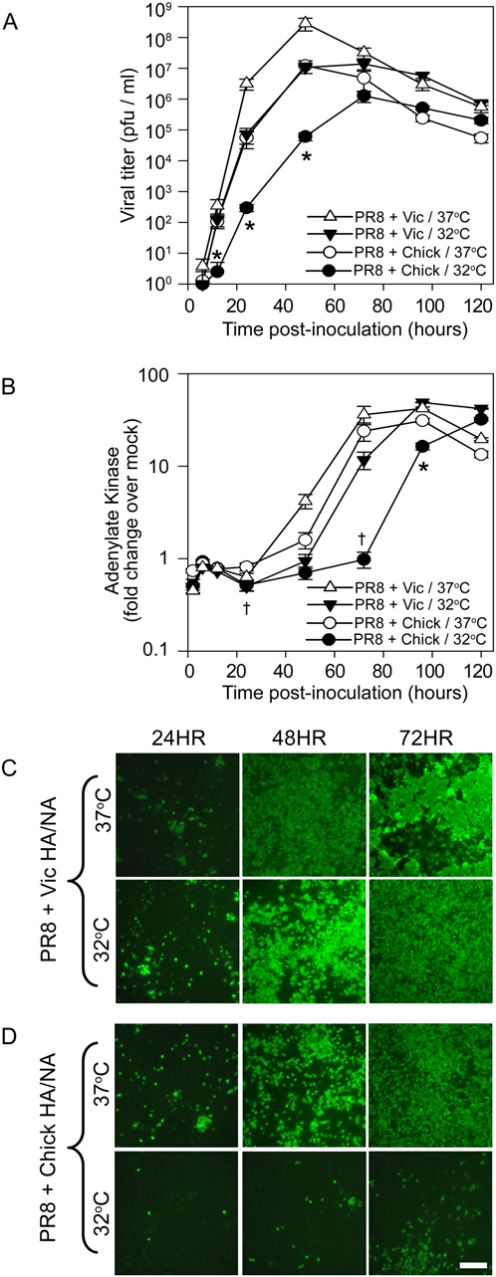
Temperature restriction of avian influenza viruses at 32°C can be mimicked by inserting avian envelope glycoproteins into human influenza viruses. (A) Multi-step growth kinetics initiated in HAE over time with PR8+Vic HA/NA at 32°C (*closed triangles*) or 37°C (*open triangles*) and PR8+Chick HA/NA at 32°C (*closed circles*) or 37°C (*open circle*s) in HAE. Apical viral titers were determined at the times shown by standard plaque assay. Data shown represents mean titer across 4–8 cultures +/−SE. (B) Adenylate kinase activity in apical washes of virus-infected HAE expressed as fold-change over adenylate kinase activity in mock-inoculated HAE +/−SE (n = 4–8). Significance is noted (*p<0.05) where viral titers or AK levels obtained for PR8+Chick HA/NA at 32°C were statistically different from all other titers/AK measurements (Chick/37°C, Vic/32°C and Vic/37°C) at that particular time point. Significance is noted (^†^p<0.05) where AK levels obtained for PR8+Chick HA/NA at 32°C and 37°C were statistically different. (C,D) Representative *en face* photomicrographs of viral nucleoprotein immunoreactivity (*green*) in HAE inoculated with (C) PR8+Vic HA/NA or (D) PR8+Chick HA/NA, at 24, 48 and 72 hrs pi at 32°C (lower rows) or 37°C (upper rows).

Growth kinetics in HAE of PR8+Vic HA/NA and PR8+Chick HA/NA inoculated at equal MOI (0.01) revealed that PR8+Vic HA/NA infection and growth was efficient at both 32°C and 37°C (Figure 6A). PR8+Chick HA/NA grew at 37°C to identical titers as PR8+Vic HA/NA at 32°C recapitulating our data obtained for wholly human (A/Victoria/3/75) and wholly avian (A/Dk/Eng/62) viruses. In contrast, PR8+Chick HA/NA was severely delayed in growth at 32°C and generated titers that were >2 logs less than titers obtained for this virus at 37°C at both 24 and 48 hrs pi. Indeed, PR8+Chick HA/NA, like A/Dk/Eng/62 avian influenza virus (Figure 1A), was significantly restricted for growth at 32°C at 12, 24 and 48 hrs pi compared to growth at 37°C and growth of PR8+Vic HA/NA at either temperature.

As observed for wholly human and avian influenza viruses, peak titers were reached for PR8+Vic HA/NA at both temperatures and PR8+Chick HA/NA at 37°C by 48 hrs pi after which a decline in viral titer was apparent. Again, as noted in our observations with human and avian influenza viruses, the loss of viral titers with time correlated with the onset of CPE. While PR8+Chick HA/NA infection at 32°C did not result in substantial AK release until 96 hr pi, increased AK activity was detected in cultures inoculated with this virus at 37°C. AK activity measured in cultures at this temperature increased with similar kinetics and reached similar levels as AK measured in cultures inoculated with PR8+Vic HA/NA at either temperature. Furthermore, the kinetics of AK induction demonstrated that again, AK was consequential to viral replication and that, overall, CPE induced by reassortant viruses was reflective of CPE measured for human and avian influenza viruses.

*En face* staining of HAE at 24 hr intervals after inoculation showed PR8+Chick HA/NA spread to additional target cells at 37°C at a rate similar to that of PR8+Vic HA/NA at 32°C and correlated with the titers measured for these two viruses under those conditions (Figure 6C and 6D). At 32°C, however, PR8+Chick HA/NA spread was severely compromised and resembled the infection characteristics shown for A/Dk/Eng/62 (H4N6) in Figure 2A. Thus, by replacing human glycoproteins with those from an avian virus isolate, we have recapitulated the effect of temperature on infection and growth kinetics as well as the degree of cytotoxicity produced by wholly avian influenza virus interactions in human ciliated airway epithelium. The relative contributions of reduced cell-cell spread and reduced CPE by avian-like influenza viruses at temperatures of the proximal airways to *in vivo* infection and pathology will, however, require further investigation.

## Discussion

We have performed comparative studies of the infection kinetics of human and avian influenza viruses in a model of human ciliated airway epithelium at temperatures reflective of the human proximal and distal airways. Our data show that avian and avianized influenza viruses are restricted for infection and growth in HAE at 32°C but not 37°C, while human viruses infect and grow efficiently at both temperatures. Based on these data, we suggest that while the warmer temperatures of the distal airways enable comparable infection by both human and avian influenza viruses, the cooler temperatures of the human proximal airways only support efficient and robust infection of the ciliated airway epithelium by human influenza viruses. We speculate that the observed restriction for avian and ‘avianized’ viruses in HAE would render avian influenza viruses more susceptible to innate and adaptive immune responses that limit pathogenicity *in vivo*. These results have significant impact on our understanding of why avian influenza viruses rarely undergo zoonotic transmission and why, when the rare human case does occur, that avian influenza virus infection and pathology manifest predominately in the warmer distal airways and lungs.

The inability of avian influenza viruses to replicate efficiently at cooler temperatures has been linked to the viral polymerase subunit, PB2 [23],[24]. In the present study, mutating position 627 in a human virus PB2 to an avian virus conserved residue resulted in growth restriction at both 32°C and 37°C, suggesting that this residue is important for general viral fitness in HAE, but is not responsible for the differences in infection seen at 32°C vs. 37°C. Two recent reports also found that viruses with 627E in PB2 were attenuated regardless of temperature in human bronchial epithelial cells and MDCK cells, respectively, although in other cell systems including human small airway epithelial cells, a temperature specific effect was found [24],[42]. It should be emphasized that those studies were performed in non-differentiated epithelial cells unlike our studies that use human differentiated airway epithelial cells. We and others have previously shown that differentiated airway epithelial cell models enable discrimination of attenuated phenotypes of respiratory virus infection whereas non-differentiated cells do not [26],[27],[43]. In addition, we also show using HAE, that the H5N1 strain A/VN/1203/04, which possesses a lysine at position 627 (human adaptation), is still restricted for growth at 32°C, albeit less so than avian influenza viruses that have never infected humans. The attenuation in HAE of this H5N1 isolate which possesses a “human” amino acid at residue 627 in PB2 suggests other residues in the polymerase subunit or other viral proteins altogether are involved in temperature sensitivity of avian influenza viruses.

In our initial experiments, spread of avian influenza viruses from cell to cell at 32°C was compromised in cultures inoculated at low MOI, suggesting a potential role for the envelope glycoproteins, HA and NA, in mediating temperature restriction. Previous work by Kaverin and colleagues also demonstrated temperature effects on growth of human-avian reassortant viruses containing avian glycoproteins [25], although this work was performed in non-polarized MDCK cells and did not investigate additional correlates of infection such as spread and CPE. In our study, we generated recombinant influenza viruses based on the A/Victoria/3/75 or A/PR/8/34 genetic backbone that were engineered to contain avian-like and/or avian glycoproteins and characterized infection in HAE. Kinetic studies showed that although human influenza viruses that possessed avian or avian-like surface glycoproteins were modestly restricted compared to wild-type viruses at 37°C, these mutant viruses were able spread like wild-type viruses throughout HAE at this temperature. Wide-spread infection throughout HAE was even observed for viruses in which their endogenous HA was replaced or mutated to preferentially bind α2,3 SA, restricting tropism to ciliated cells. Efficient replication of Vic-226-228HA at 37°C in our studies corroborates previous work by Matrosovich and colleagues in which little effect of HA-specificity ‘switching’ on replication was noted unless a very low MOI (0.00004) was used for inoculation [44]. In contrast, Wan and Perez described more profound differences in replication in HAE at 37°C with recombinant viruses that differed only in their receptor specificity [31]. However, it should be noted that their recombinant viruses were based on an H9N2 avian strain that yielded relatively low titers, and their initial infections were performed at 35°C before incubating at 37°C [31].

Compared to 37°C, viruses with a preference for binding to α2,3 SA, including Vic-226-228HA, were restricted for growth and spread in HAE at 32°C. Notably, the H5N1 strain examined in this study also maintains preference for α2,3 SA binding [45]; thus, we may surmise that this characteristic of A/VN/1203/04 contributes to its attenuation observed in HAE. The contribution of α2,3 SA usage to replication of influenza viruses investigated by Hatta et al. in the upper respiratory tract of mice may have been masked in the mouse model (the 627 mutation in PB2 being more apparent) as mice express solely avian virus-like receptors (α2,3 SA) in their airways [46]. Restriction of α2,3 SA-binding viruses in HAE at 32°C was not due to a discrepancy in SA expression since HAE maintained at either 32°C or 37°C expressed similar levels of α2,6 and α2,3 SA (as detected by *Sambucus nigra* (SNA) and *Maackia amurensis* (MAA) lectin staining, respectively; data not shown).

In conjunction with the HA, the sialidase activity of NA is crucial for successful virus penetration of mucus layers for initial infection and subsequent release of progeny virions from infected cells [47],[48]. This is especially critical both *in vivo* and in HAE models in which the luminal epithelial cell surface is robust with glycoconjugates displaying abundant terminal sialic acid moieties that may act as false receptors for influenza viruses [49]. Using standard laboratory assays that employ small monovalent soluble substrates for cleavage by NA (MUNANA), we were not able to demonstrate any temperature-dependent loss of NA activity associated with either human or avian virus (data not shown). However, the ability of the avian virus NA to cleave biologically relevant substrates present in HAE may be compromised at 32°C vs. 37°C restricting both initial infection and subsequent spread of the virus throughout the epithelium. This is supported by our data which demonstrate restricted growth and spread of reassortant viruses containing avian virus NA, including Vic+Chick N1 and PR8+Chick HA/NA in HAE at 32°C.

In addition to their independent functions, the balance between the binding affinity of the viral HA and the sialidase activity of the NA is also critical for efficient infection. The ability of A/Victoria/3/75 viruses with mutations or substitutions in either the HA or NA alone to infect similar numbers of cells and replicate to comparable peak titers as for wild-type virus at 37°C implies that these viruses were not crippled by the mismatch between the specificities of their HA and NA. Replication and spread of influenza viruses that possess an avian HA paired with its “matched” NA was even more compromised than that of recombinant viruses with individual changes to levels seen with wholly avian viruses. Thus, viruses with co-evolved glycoprotein pairs exhibit restricted replication at low temperatures and both HA and NA genes contribute to the phenotype.

Together, these data imply that in the complex environment of the luminal surface of the human ciliated airway epithelium, the viral surface antigens have a marked effect on the extent of virus infection and that temperature plays an important role in limiting avian, but not human, influenza virus infection and spread in the cooler proximal airway regions. Given these results, we draw attention to other recently published data using the HAE model in which mutations in viruses that are growth attenuated *in vivo* display similar growth attenuation in HAE but not in non-differentiated cell lines, suggesting that HAE possess discriminating properties of attenuating phenotypes of mutants of respiratory viruses [26],[27]. Admittedly, in the present study, despite restriction in both growth and spread, wild-type avian viruses and human viruses with avian or avian-like glycoproteins did eventually reach high titer at 32°C at later time points. The efficiency of infection and replication of a virus that inoculates the airway epithelium, however, is likely a critical factor in determining whether the virus is capable of establishing infection in a host that normally possesses innate and adaptive immune systems that attempt to limit virus infection and spread. At temperatures of the distal airways, avian influenza viruses displayed similar infection kinetics as human influenza viruses and would therefore, in the case of sufficient inoculum reaching these distal regions, be as likely to establish infection. Indeed, the clinical pathology findings for humans infected with H5N1 do report distal airway infection in ciliated bronchioles and lung regions [22]. Under these conditions of inoculation and infection, avian influenza viruses present in the distal airways may still be unable to spread to proximal airway regions without additional adaptation to cooler temperatures. One caveat of this prediction is that virus may be transported to proximal airway regions by innate mucus clearance mechanisms indicating that caution is required when attempting to identify proximal infection by viruses in airway secretions obtained from tracheal swabs.

In conclusion, the present study substantiates differential host temperature as a critical barrier for infection by avian influenza viruses. Since the ciliated airway epithelium of the proximal airways is a major portal for influenza virus infection and spread, accessible by multiple inoculation routes (e.g., ocular, nasopharyngeal or aerosol), the inability of avian influenza viruses to establish infection and spread in these regions would be predicted to reduce the frequency of successful zoonotic transmission. Furthermore, the ability of human influenza viruses to generate high viral titers in the human proximal airways is likely a factor in effective human-to-human transmission and the induction of airway epithelial cell cytotoxicity as shown in this study may increase particulate matter perhaps associated with virus that facilitates inoculation of new hosts. Rapid induction of cytotopathic effects by human, but not avian, influenza virus infection at the temperature of the human proximal airways may also contribute to the onset of other host defenses such as sneezing and coughing that facilitate clearance of particulate matter/virus from the airways and potentially promote transmission between human hosts.

## Materials and Methods

### Cells

Human airway tracheobronchial epithelial cells isolated from airway specimens from patients without underlying lung disease were provided by the National Disease Research Interchange (NDRI, Philadelphia, PA) or as excess tissue following lung transplantation under University of North Carolina at Chapel Hill (UNC) Institutional Review Board-approved protocols by the UNC Cystic Fibrosis Center Tissue Culture Core. Primary cells derived from single patient sources were expanded on plastic to generate passage 1 cells and plated at a density of 3×10^5^ cells per well on permeable Transwell-Col (12-mm diameter) supports (Corning, Inc.). HAE cultures were grown in custom media with provision of an air-liquid interface for 4 to 6 weeks to form differentiated, polarized cultures that resemble *in vivo* pseudostratified mucociliary epithelium, as previously described [50]. Madin-Darby Canine Kidney (MDCK) cells were maintained in DMEM (Gibco-Invitrogen, Inc.) supplemented with 10% fetal bovine serum and 1% penicillin / streptomycin (Sigma-Aldrich, Inc.).

### Viruses

Influenza virus A/England/26/99 (H3N2) was isolated at the Health Protection Agency, Colindale, London, UK, during the routine surveillance program and has been minimally passaged in MDCK cells [51]. A/Dk/Singapore/97 (H5N3) and A/Dk/England/62 (H4N6) are typical avian influenza strains that have been passaged in both embryonated chicken eggs and MDCK cells during laboratory handling. Highly pathogenic A/VN/1203/04 (H5N1) was biologically derived and minimally passaged in embryonated chicken eggs. A/Udorn/307/72 (H3N2) was passed in baby hamster kidney (BHK) cells and represents a clone expanded once in embryonated chicken eggs. Recombinant viruses, including wild-type A/Victoria/3/75 (H3N2) and mutants in either the A/Victoria/3/75 (H3N2) or A/PR/8/24 (H1N1) background, were generated from cloned cDNA in 293T and MDCK cell co-cultures as previously described [52],[53]. Mutant viruses were generated in either the A/Victoria/3/75 (H3N2) or A/PR/8/34 (H1N1) genetic background as follows: 1) Vic 627PB2; A/Victoria/3/75 containing a lysine to glutamic acid amino acid substitution at position 627; 2) Vic-226-228HA; A/Victoria/3/75 containing two amino acid substitutions in the HA gene (L226Q, S228G) that confer an avian-like receptor binding preference [6],[40]; 3) Vic+Chick N1; A/Victoria/3/75 in which segment 6 containing the endogenous N2 NA gene was exchanged for the N1 NA gene from avian isolate A/Chick/Italy/1347/99; 4) Vic-226-228HA+Chick N1; A/Victoria/3/75 containing both L226Q and S228G mutations and the avian N1; 5) PR8+Vic HA/NA; A/PR/8/34 in which the endogenous H1 and N1 were replaced with the H3 and N2 from A/Victoria/3/75 and 6) PR8+Chick HA/NA (RD3); A/PR/8/34 in which the endogenous H1 and N1 were replaced with the H7 and N1 from A/Chick/Italy/1347/99. (RD3 was previously described as a candidate vaccine strain [41].) The last two reassortant viruses were generated by substituting segment 4 and segment 6 from PR8 with those from either A/Victoria/3/75 (H3N2) or A/Chick/Italy/1347/99 (H7N1). The multi-basic cleavage site in the avian H7 HA gene used in these studies was removed prior to rescue of these recombinant viruses for safety. Available accession numbers (GenBank: http://www.ncbi.nlm.nih.gov.libproxy.lib.unc.edu) are V01086 for A/Victoria/3/75 HA and CAD37074 for A/Chick/Italy/1347/99 HA.

### Viral inoculation and growth in HAE

HAE were rinsed with PBS to transiently remove apical secretions and supplied with fresh basolateral medium prior to inoculation. Virus inoculum was diluted in PBS and applied to the apical surface of HAE for 2 hrs at either 32°C, 33°C, or 37°C, as indicated. Following incubation, viral inocula were removed and cultures incubated at 32°C, 33°C or 37°C for the duration of the experiment. Viral growth kinetics were determined by performing apical washes with 300 µl of serum-free DMEM for 30 min at either 32°C or 37°C. Washes were harvested and stored at −80°C prior to analysis. Viral titers in the apical washes were determined by standard plaque assay or tissue culture infectious dose (TCID)_50_ assay on MDCK cell monolayers as previously described [13],[52],[54].

### En face staining

At various points post-inoculation (pi), HAE were fixed in cold methanol-acetone (50/50) and stored at 4°C. Cultures were then permeabilized with 2.5% triton-X 100/PBS++ (containing 1 mM CaCl_2_ and 1 mM MgCl_2_) and blocked with 3% bovine serum albumin (BSA) in PBS++ before being probed with mouse anti-influenza virus nucleoprotein (NP; Chemicon, Inc.; 1∶100) and immunoreactivity detected with fluorescein isothiocyanate (FITC)-conjugated anti-mouse IgG secondary antibody (Jackson ImmunoResearch Laboratories, Inc., 1∶500). Fluorescent images were obtained using a Leica DMIRB inverted fluorescence microscope equipped with cooled-color charge-coupled-device digital camera (MicroPublisher; Q-Imaging, Burnaby, BC, Canada). The percentage of the epithelium positive for viral antigen as an index of percentage of infected cells was quantified over 5 images per culture by black and white pixilation of each image and computer calculation of percent black pixels after inverting the image. This technique determines percentage of black pixels in a defined area and does not account for differences in fluorescent intensity.

### Measures of CPE

Viral-induced cytotoxicity was determined by measuring adenylate kinase activity in apical washes using a commercially available assay (Lonza, Inc.). Apical samples were centrifuged prior to freezing to remove any cellular contaminants present in the wash. Luminescence detected in samples from infected HAE were normalized to uninfected HAE and expressed as fold change over AK measured in uninfected (mock) HAE. Morphological assessment of cytotoxicity in HAE was performed with paraformaldehyde (PFA, 4%)-fixed histological sections (5 µm) stained with hematoxylin and eosin.

### Detection of α2,3 and α2,6 linked sialic acids

HAE maintained at either 32°C or 37°C for 72 hrs prior to sialic acid detection were washed, blocked with 3% BSA/PBS++ and probed with biotinylated SNA or MAA lectins to detect α2,6 and α2,3 SA, respectively (Vector Laboratories, Inc.; EY-Laboratories, Inc.; 1∶100). HAE were then fixed in 4% PFA and incubated with streptavidin-alexafluor 488 (Molecular Probes, Inc.; 1∶500) applied to the apical surface to detect lectin binding.

### Immunohistochemistry

HAE fixed in methanol∶acetone, were probed *en face* with antibody against viral NP (Chemicon, Inc.; 1∶100) and FITC-conjugated goat anti-mouse IgG1 and IgG2a (Jackson ImmunoResearch Laboratories, Inc., West Grove, PA; 1∶500), then embedded in paraffin. Histological sections (5 µm) were prepared and reprobed for viral antigen using standard immunofluorescence protocols. Briefly, sections were bathed in 2.5% triton-X 100/PBS++ for 30 min, blocked in 3% BSA/PBS++ and incubated with antibodies in 1% BSA/PBS++. Primary antibodies were anti-viral NP (Chemicon, Inc., as above) and anti-alpha acetylated tubulin (Zymed Laboratories, Inc.; 1∶2000), a marker for ciliated cells. Secondary antibodies were FITC-goat anti-mouse IgG2a and Rhodamine red-conjugated goat-anti-mouse IgG2b (Jackson ImmunoResearch Laboratories, Inc.; 1∶500). Sections were prepared with FluorSave mounting media (EMD Chemicals, Inc.) and images captured using a Leica DMIRB inverted fluorescence microscope equipped with a cooled color charge-coupled-device digital camera (MicroPublisher; Q-Imaging, Burnaby, British Columbia, Canada).

### Statistical analysis

Linear mixed models were fitted to the repeated measurements of log-transformed viral titer over time that included effects for the four treatment groups (defined by virus and temperature), eight time points, and the interaction between treatment and time. We note that in a small number of cases, there were only two treatment groups (defined by temperature) and fewer than eight time points. A heterogeneous autoregressive correlation structure of order one was assumed for the repeated measurements. A joint test of the interaction terms (21 degrees of freedom) provides an assessment of the hypothesis of no differences among the four treatment groups with respect to viral titer growth (log scale). Provided this test was significant, indicating some differences among the four growth curves, pair-wise differences between the three treatment groups versus the a priori specified reference group (generally the avian strain at the lowest temperature) were carried out for each time point, and significant differences at the 0.05 level were noted. No adjustments for inflated Type I error due to multiple comparisons were made. Missing observations were assumed to be missing completely at random, based on the fact that the investigators determined a priori to remove samples at specific time points during the experiment.

## References

[1] Prevention CfDCa (2008). Update: Influenza Activity-United States, September 30, 2007–February 9, 2008.. MMWR.

[2] Webster RG, Bean WJ, Gorman OT, Chambers TM, Kawaoka Y (1992). Evolution and ecology of influenza A viruses.. Microbiol Rev.

[3] Horimoto T, Kawaoka Y (2005). Influenza: lessons from past pandemics, warnings from current incidents.. Nat Rev Microbiol.

[4] Reid AH, Taubenberger JK, Fanning TG (2004). Evidence of an absence: the genetic origins of the 1918 pandemic influenza virus.. Nat Rev Microbiol.

[5] Scholtissek C, Rohde W, Von Hoyningen V, Rott R (1978). On the origin of the human influenza virus subtypes H2N2 and H3N2.. Virology.

[6] Connor RJ, Kawaoka Y, Webster RG, Paulson JC (1994). Receptor specificity in human, avian, and equine H2 and H3 influenza virus isolates.. Virology.

[7] Gambaryan AS, Tuzikov AB, Piskarev VE, Yamnikova SS, Lvov DK (1997). Specification of receptor-binding phenotypes of influenza virus isolates from different hosts using synthetic sialylglycopolymers: non-egg-adapted human H1 and H3 influenza A and influenza B viruses share a common high binding affinity for 6′-sialyl(N-acetyllactosamine).. Virology.

[8] Matrosovich MN, Gambaryan AS, Teneberg S, Piskarev VE, Yamnikova SS (1997). Avian influenza A viruses differ from human viruses by recognition of sialyloligosaccharides and gangliosides and by a higher conservation of the HA receptor-binding site.. Virology.

[9] Rogers GN, Paulson JC (1983). Receptor determinants of human and animal influenza virus isolates: differences in receptor specificity of the H3 hemagglutinin based on species of origin.. Virology.

[10] Shinya K, Ebina M, Yamada S, Ono M, Kasai N (2006). Avian flu: influenza virus receptors in the human airway.. Nature.

[11] Nicholls JM, Bourne AJ, Chen H, Guan Y, Peiris JS (2007). Sialic acid receptor detection in the human respiratory tract: evidence for widespread distribution of potential binding sites for human and avian influenza viruses.. Respir Res.

[12] Nicholls JM, Chan MC, Chan WY, Wong HK, Cheung CY (2007). Tropism of avian influenza A (H5N1) in the upper and lower respiratory tract.. Nat Med.

[13] Thompson CI, Barclay WS, Zambon MC, Pickles RJ (2006). Infection of human airway epithelium by human and avian strains of influenza a virus.. J Virol.

[14] Matrosovich MN, Matrosovich TY, Gray T, Roberts NA, Klenk HD (2004). Human and avian influenza viruses target different cell types in cultures of human airway epithelium.. Proc Natl Acad Sci U S A.

[15] Matrosovich M, Zhou N, Kawaoka Y, Webster R (1999). The surface glycoproteins of H5 influenza viruses isolated from humans, chickens, and wild aquatic birds have distinguishable properties.. J Virol.

[16] Stevens J, Blixt O, Tumpey TM, Taubenberger JK, Paulson JC (2006). Structure and receptor specificity of the hemagglutinin from an H5N1 influenza virus.. Science.

[17] Tumpey TM, Maines TR, Van Hoeven N, Glaser L, Solorzano A (2007). A two-amino acid change in the hemagglutinin of the 1918 influenza virus abolishes transmission.. Science.

[18] McFadden ER, Pichurko BM, Bowman HF, Ingenito E, Burns S (1985). Thermal mapping of the airways in humans.. J Appl Physiol.

[19] Lindemann J, Leiacker R, Rettinger G, Keck T (2002). Nasal mucosal temperature during respiration.. Clin Otolaryngol Allied Sci.

[20] Hayden F, Croisier A (2005). Transmission of avian influenza viruses to and between humans.. J Infect Dis.

[21] Bitko V, Musiyenko A, Barik S (2007). Viral infection of the lungs through the eye.. J Virol.

[22] Uiprasertkul M, Puthavathana P, Sangsiriwut K, Pooruk P, Srisook K (2005). Influenza A H5N1 replication sites in humans.. Emerg Infect Dis.

[23] Massin P, van der Werf S, Naffakh N (2001). Residue 627 of PB2 is a determinant of cold sensitivity in RNA replication of avian influenza viruses.. J Virol.

[24] Hatta M, Hatta Y, Kim JH, Watanabe S, Shinya K (2007). Growth of H5N1 influenza A viruses in the upper respiratory tracts of mice.. PLoS Pathog.

[25] Kaverin NV, Rudneva IA, Smirnov YA, Finskaya NN (1988). Human-avian influenza virus reassortants: effect of reassortment pattern on multi-cycle reproduction in MDCK cells.. Arch Virol.

[26] Bartlett EJ, Hennessey M, Skiadopoulos MH, Schmidt AC, Collins PL (2008). The role of interferon in the replication of human parainfluenza virus type 1 wild type and mutant viruses in human ciliated airway epithelium.. J Virol.

[27] Bartlett EJ, Cruz AM, Esker J, Castano A, Schomacker H (2008). Human parainfluenza virus type 1 C proteins are non-essential proteins that inhibit the host interferon and apoptotic responses and are required for efficient replication in non-human primates.. J Virol.

[28] Zhang L, Bukreyev A, Thompson CI, Watson B, Peeples ME (2005). Infection of ciliated cells by human parainfluenza virus type 3 in an in vitro model of human airway epithelium.. J Virol.

[29] Zhang L, Peeples ME, Boucher RC, Collins PL, Pickles RJ (2002). Respiratory syncytial virus infection of human airway epithelial cells is polarized, specific to ciliated cells, and without obvious cytopathology.. J Virol.

[30] Sims AC, Baric RS, Yount B, Burkett SE, Collins PL (2005). Severe acute respiratory syndrome coronavirus infection of human ciliated airway epithelia: role of ciliated cells in viral spread in the conducting airways of the lungs.. J Virol.

[31] Wan H, Perez DR (2007). Amino acid 226 in the hemagglutinin of H9N2 influenza viruses determines cell tropism and replication in human airway epithelial cells.. J Virol.

[32] Chen LM, Davis CT, Zhou H, Cox NJ, Donis RO (2008). Genetic compatibility and virulence of reassortants derived from contemporary avian H5N1 and human H3N2 influenza A viruses.. PLoS Pathog.

[33] Hers JF (1966). Disturbances of the ciliated epithelium due to influenza virus.. Am Rev Respir Dis.

[34] Maines TR, Lu XH, Erb SM, Edwards L, Guarner J (2005). Avian influenza (H5N1) viruses isolated from humans in Asia in 2004 exhibit increased virulence in mammals.. J Virol.

[35] Lam WY, Tang JW, Yeung AC, Chiu LC, Sung JJ (2008). Avian influenza virus A/HK/483/97(H5N1) NS1 protein induces apoptosis in human airway epithelial cells.. J Virol.

[36] Daidoji T, Koma T, Du A, Yang CS, Ueda M (2008). H5N1 avian influenza virus induces apoptotic cell death in mammalian airway epithelial cells.. J Virol.

[37] Almond JW (1977). A single gene determines the host range of influenza virus.. Nature.

[38] Subbarao EK, London W, Murphy BR (1993). A single amino acid in the PB2 gene of influenza A virus is a determinant of host range.. J Virol.

[39] Yao Y, Mingay LJ, McCauley JW, Barclay WS (2001). Sequences in influenza A virus PB2 protein that determine productive infection for an avian influenza virus in mouse and human cell lines.. J Virol.

[40] Vines A, Wells K, Matrosovich M, Castrucci MR, Ito T (1998). The role of influenza A virus hemagglutinin residues 226 and 228 in receptor specificity and host range restriction.. J Virol.

[41] Whiteley AMD, Legastelois I, Campitelle L, Donatelli I (2007). Generation of candidate human influenza vaccine strains in cell culture—rehearsing the European response to an H7N1 pandemic threat.. Influenza Resp Viruses.

[42] Steel J, Lowen AC, Mubareka S, Palese P (2009). Transmission of influenza virus in a mammalian host is increased by PB2 amino acids 627K or 627E/701N.. PLoS Pathog.

[43] Wright PF, Ikizler MR, Gonzales RA, Carroll KN, Johnson JE (2005). Growth of respiratory syncytial virus in primary epithelial cells from the human respiratory tract.. J Virol.

[44] Matrosovich M, Matrosovich T, Uhlendorff J, Garten W, Klenk HD (2007). Avian-virus-like receptor specificity of the hemagglutinin impedes influenza virus replication in cultures of human airway epithelium.. Virology.

[45] Yen HL, Lipatov AS, Ilyushina NA, Govorkova EA, Franks J (2007). Inefficient transmission of H5N1 influenza viruses in a ferret contact model.. J Virol.

[46] Ibricevic A, Pekosz A, Walter MJ, Newby C, Battaile JT (2006). Influenza virus receptor specificity and cell tropism in mouse and human airway epithelial cells.. J Virol.

[47] Matrosovich MN, Matrosovich TY, Gray T, Roberts NA, Klenk HD (2004). Neuraminidase is important for the initiation of influenza virus infection in human airway epithelium.. J Virol.

[48] Gottschalk A (1957). Neuraminidase: the specific enzyme of influenza virus and Vibrio cholerae.. Biochim Biophys Acta.

[49] Stonebraker JR, Wagner D, Lefensty RW, Burns K, Gendler SJ (2004). Glycocalyx restricts adenoviral vector access to apical receptors expressed on respiratory epithelium in vitro and in vivo: role for tethered mucins as barriers to lumenal infection.. J Virol.

[50] Pickles RJ, McCarty D, Matsui H, Hart PJ, Randell SH (1998). Limited entry of adenovirus vectors into well-differentiated airway epithelium is responsible for inefficient gene transfer.. J Virol.

[51] Thompson CI, Barclay WS, Zambon MC (2004). Changes in in vitro susceptibility of influenza A H3N2 viruses to a neuraminidase inhibitor drug during evolution in the human host.. J Antimicrob Chemother.

[52] Elleman CJ, Barclay WS (2004). The M1 matrix protein controls the filamentous phenotype of influenza A virus.. Virology.

[53] Neumann G, Watanabe T, Ito H, Watanabe S, Goto H (1999). Generation of influenza A viruses entirely from cloned cDNAs.. Proc Natl Acad Sci U S A.

[54] Gaush CR, Smith TF (1968). Replication and plaque assay of influenza virus in an established line of canine kidney cells.. Appl Microbiol.

